# Expression of TRAF6 and ubiquitin mRNA in skeletal muscle of gastric cancer patients

**DOI:** 10.1186/1756-9966-31-81

**Published:** 2012-09-26

**Authors:** Yuan-Shui Sun, Zai-Yuan Ye, Zhen-Yuan Qian, Xiao-Dong Xu, Jun-Feng Hu

**Affiliations:** 1 Departments of Gastrointestinal Surgery, Zhejiang Provincial People’s Hospital, Hangzhou, 310014, PR China

**Keywords:** Gastric cancer, TRAF6, Ubiquitin, Mrna, Skeletal muscle, Cachexia

## Abstract

**Objective:**

To investigate the prognostic significance of tumor necrosis factor receptor (TNFR),-associated factor 6 (TRAF6),-and ubiquitin in gastric cancer patients.

**Methods:**

Biopsies of the rectus abdominis muscle were obtained intra operatively from 102 gastric cancer patients and 29 subjects undergoing surgery for benign abdominal diseases, and muscle TRAF6 and ubiquitin mRNA expression and proteasome proteolytic activities were assessed.

**Results:**

TRAF6 was significantly upregulated in muscle of gastric cancer compared with the control muscles. TRAF6 was upregulated in 67.65% (69/102) muscle of gastric cancer. Over expression of TRAF6 in muscles of gastric cancer were associated with TNM stage, level of serum albumin and percent of weight loss. Ubiquitin was significantly upregulated in muscle of gastric cancer compared with the control muscles. Ubiquitin was upregulated in 58.82% (60/102) muscles of gastric cancer. Over expression of ubiquitin in muscles of gastric cancer were associated with TNM (Tumor-Node-Metastasis) stage and weight loss. There was significant relation between TRAF6 and ubiquitin expression.

**Conclusions:**

We found a positive correlation between TRAF6 and ubiquitin expression, suggesting that TRAF6 may up regulates ubiquitin activity in cancer cachexia. While more investigations are required to understand its mechanisms of TRAF6 and ubiquitin in skeletal muscle. Correct the catabolic-anabolic imbalance is essential for the effective treatment of cancer cachexia.

## Introduction

Cancer cachexia is a complex metabolic condition characterized by loss of skeletal muscle. Common clinical manifestations include muscle wasting, anemia, reduced caloric intake, and altered immune function, which contribute to increased disability, fatigue, diminished quality of life, and reduced survival [1-3]. Many patients with cancer present with weight loss at diagnosis, and much of this weight loss can be attributed to muscle wasting. Cancer cachexia has been viewed as an end-of-life condition in patients with advanced or incurable malignancies that was managed primarily through palliative approaches. However, cachexia and associated skeletal muscle loss may be present early in the progression of cancer, indicating the importance of earlier diagnosis and treatment.

The prevalence of cancer cachexia varies depending on the type of malignancy, with the greatest frequency of weight loss (50%–85% of patients) observed in gastrointestinal, pancreatic, lung, and colorectal cancers at diagnosis and before initiation of chemotherapy [4]. One common mechanism associated with skeletal muscle protein degradation in cancer cachexia is the activation of the adenosine triphosphate-dependent ubiquitin-proteasome proteolytic path way [5,6]. This system plays a major role in muscle wasting and, more specifically, in the breakdown of myofibrillar proteins. Certainly, the mechanisms of muscle wasting in cancer cachexia are complex. They involve multiple host and tumor factors, decreased levels of testosterone and insulin-like growth factor-1 (IGF-1), and decreased food intake, contributing to both antianabolic and procatabolic processes [7,8]. The study demonstrate that the expression level of tumor necrosis factor (α) receptor adaptor protein 6 (TRAF6), a protein involved in receptor-mediated activation of several signaling pathways, is enhanced in skeletal muscle during atrophy [9,10].

Here, we analyzed the prognostic significance of tumor necrosis factor receptor (TNFR)-associated factor 6 (TRAF6), and ubiquitin in gastric cancer patients. The current study were to examine the expression of TRAF6 and ubiquitin in skeletal muscle specimens of patients with gastric cancer, to explore the possible correlation among TRAF6, ubiquitin mRNA expression and cachexia.

## Methods

### Patients and tissue samples

Skeletal muscle tissues were collected from one hundred and two patients with gastric cancer (median age 61.0y, range 42–88y; 24 male, 10 female) from the Department of Surgery, Zhejiang Provincial People’s Hospital from January 2008 to January 2011. Patients’ characteristics are showed in Table 1. Diagnosis of gastric cancer was performed by endoscopic biopsy. Twenty-nine patients undergoing surgery for benign abdominal diseases served as a control group, there were 12 cholelithiasis, 9 inguinal hernia, 8 hemangioma of liver. Gastric cancer patients and controls were similar in terms of age and sex distribution. Nevertheless, gastric cancer patients showed a significantly lower body mass index, serum albumin levels and prognostic nutritional index. Exclusion criteria for both groups were considered: acute or chronic renal failure, liver failure, diabetes, metabolic acidosis, sepsis, AIDS, inflammatory bowel disease, autoimmune disorders, chronic heart failure, and hyperthyroidism. The study was approved by our hospital ethics committees. Written informed consent for the study procedures was obtained from the patients.

**Table 1:** Summary of characteristics of gastric cancer patients and control

	**Controls (n = 29)**	**Gastric cancer (n = 102)**	**t/*χ***^***2***^	**P Value**
Age, y	61.88 ± 6.49	62.13 ± 6.54	0.053	0.959
Sex (M:F)	21:8	72:30	0.037	0.848
Weight loss	65.50 ± 4.84	57.38 ± 6.28	2.899	0.012
BMI	24.13 ± 1.81	21.00 ± 1.31	3.96	0.001
Serum albumin, g/L	41.38 ± 6.09	33.75 ± 3.11	3.15	0.007
PNI	45.25 ± 3.62	37.18 ± 3.74	5.26	0.0001

### Nutritional assessment

The nutritional assessment included anthropometric [height, actual body weight, %WL, body mass index (BMI), usual body weight], immunological (total lymphocyte count), and biochemical (serum albumin) indexes. Routine blood test was determined using completely automatic blood cell count analyzer (Beckman-Coulter -MAXM, American). Liver function was determined using Completely automatic biochemistry analyzer (Beckman-Coulter SYNCHRON LX 20, American) (Table 1). The PNI(prognostic nutritional index) was calculated as follows: PNI = 10 × serum albumin(g/100 ml) + 0.005 × total lymphocyte count/mm^3^ of peripheral blood [11].

### Muscle biopsy

A biopsy specimen was obtained from the rectus abdominis muscle during the initial phase of the operation. The anterior sheet of the rectus abdominis muscle was opened with scissors after skin incision and dissection through the subcutaneous fat, and a muscle biopsy specimen weighing about 1.0 g was obtained. The biopsy specimen was divided into two portions that were immediately frozen in liquid nitrogen and then stored at −80°C until analysis. No complications occurred from the biopsy procedure.

### Real-time quantitative RT-PCR

Total RNA from rectus abdominis muscle was extracted by TRIzol reagent and cDNAs were reverse-transcribed by Revert Aid TM reverse transcriptase. Real-time PCR was carried out using the ABI PRISM 7700 Sequence Detection System (Applied Bio systems) at 50°C for 2 min, 95°C for 10 min, followed by 50 cycles at 95°C for 15 s, and at 60°C for 1 min. The primers for GAPDH (224 bp) were 5'-TGAAGGTCGGAGTCAACGG-3' (sense) and 5'- CTGGAAGATGGTGATGGGATT-3' (antisense). The primers for TRAF6 (134 bp) were 5'-GCCTGGGTGACAGAGTGC-3' (sense) and 5'-AATGACTACTTATGGCTCCTTTTC-3' (antisense). The primers for ubiquitin(165 bp) were 5'-CCCTGGATGTGATGGTGTC-3' (sense) and 5'-CTCGTTGTCCCTGTTGCTG-3' (antisense). The expression of GAPDH was used to normalize that of the target genes. Each assay was done in triplicate, the average was calculate, and the expression level of TRAF6 and ubiquitin was expressed as 2^–ΔΔCt^, ΔCt = Ct (Target)–Ct (GAPDH).

### Immunoblotting

Cells were lysed in RIPA buffer (150 mM NaCl, 10 mM Tris, pH 7.5, 1% NP40, 1% deoxycholate, 0.1% SDS, protease inhibitor cocktail (Roche)). Total proteins were fractionated using the NuPAGE 4–12% Bis-Tris gradient gel (Invitrogen) and transferred onto PVDF membrane. Membranes were blocked with 5% non-fat milk in PBS/Tween-20, and incubated with antibodies against TRAF6 (Santa Cruz), ubiquitin (Santa Cruz), and β-actin (Abcam).

### Statistical analysis

In order to analyze the relationship among the expression of TRAF6 and ubiquitin and nutritional status of patients (percent weight loss, serum albumin), according to the literature [12], they were divided into two groups(percent weight loss ≥ 10 and <10, serum albumin ≥ 35and <35). All statistical analyses were performed using SPSS16.0 software. Measurement data were analyzed using the Student’s t test, while categorical data were studied using χ^2^ or Fisher exact tests. Statistical significance was set at P < 0.05.

## Results

### The expression of TRAF6 in muscle of control and cancer patients

**Figure 1:**
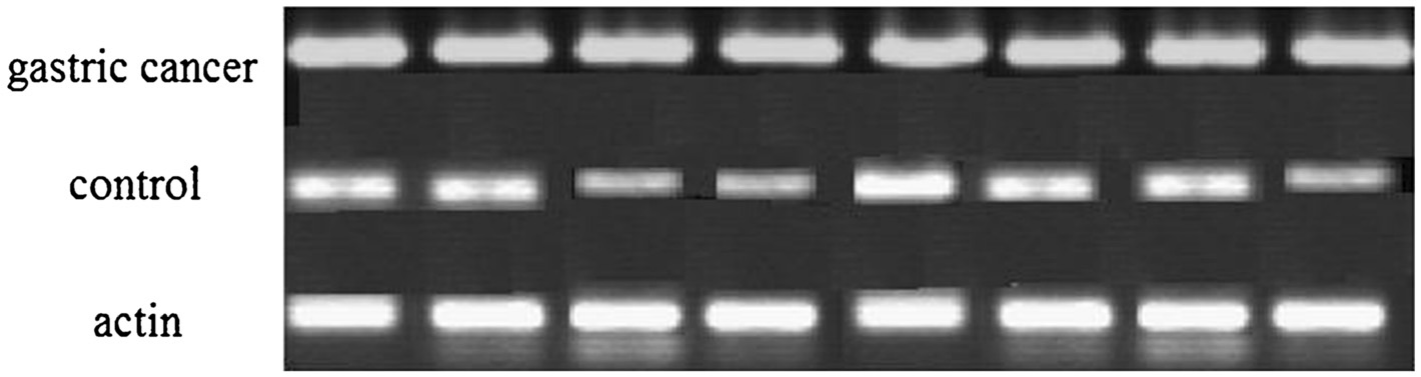
The level of TRAF6 protein in muscle of cancer patients and control.

Tumor necrosis factor (α) receptor adaptor protein 6(TRAF6), a protein involved in receptor-mediated activation of several signaling pathways, is enhanced in skeletal muscle during atrophy. We assessed the expression of TRAF6 in 29 control muscles and 102 patient muscles. TRAF6 was significantly upregulated in muscle of gastric cancer compared with the control muscles (P < 0.05). TRAF6 was upregulated in 67.65% (69/102) muscle of gastric cancer. Overexpression of TRAF6 in muscles of gastric cancer were associated with TNM stage, level of serum albumin and percent of weight loss (P > 0.05) (Table 2). We also analyze the expression of TRAF6 in 8 muscles of control and cancer patients by western blotting, the results show the expression of TRAF6 in muscle of cancer patients were higher than control (Figure 1).

**Table 2:** The expression of TRAF6 in muscle of cancer patients

	**low**	**high**	***χ***^***2***^	**P Value**
Percent weight loss			12.9	0.001
≥10	9	45		
<10	24	24		
Serum albumin,g/L			20.05	0.001
≥35	24	18		
<35	9	51		
TNM stage			13.33	0.001
I-II	21	18		
III-IV	12	51		

### The expression of ubiquitin in muscle of control and cancer patients

**Figure 2:**
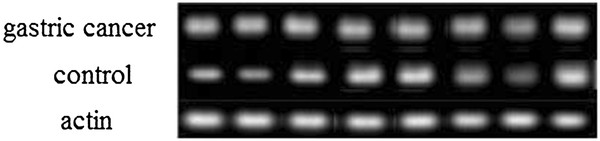
The level of ubiquitin protein in muscle of cancer patients and control.

We assessed the expression of ubiquitin in 29 control muscles and 102 patient muscles. Ubiquitin was significantly upregulated in muscle of gastric cancer compared with the control muscles (P < 0.05). Ubiquitin was upregulated in 58.82% (60/102) muscles of gastric cancer. Over expression of ubiquitin in muscles of gastric cancer were associated with TNM stage and weight loss (P > 0.05) (Table 3). In order to analyze the expression of ubiquitin protein with quantitation, 8 muscle of control and cancer patients were detectec by western blotting, the study indicated the expression of ubiquitin in 5 muscle of cancer patients were higher than control (Figure 2).

**Table 3:** The expression of ubiquitin in muscle of cancer patients

	**low**	**high**	***χ***^***2***^	**P Value**
Percent weight loss			11.78	0.001
≥10	15	42		
<10	27	18		
Serum albumin,g/L			15.74	0.001
≥35	27	15		
<35	15	45		
TNM stage			20.52	0.001
I-II	27	12		
III-IV	15	48		

### Association between expression of TRAF6 and ubiquitin

**Figure 3:**
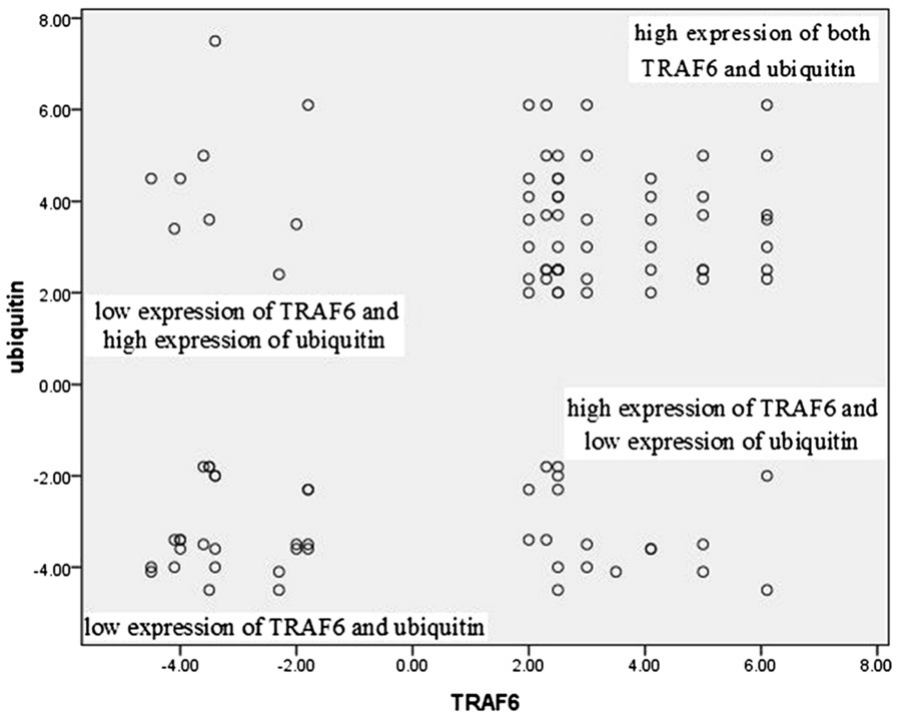
Association between expression of TRAF6 and ubiquitin.

Seventeen cases of gastric cancer had high expression of both TRAF6 and ubiquitin, and eight cases of gastric cancer had low expression of both TRAF6 and ubiquitin. There was significant between TRAF6 and ubiquitin expression (*χ*^*2*^ =6.68; *P* = 0.01) (Table 4, Figure 3).

**Table 4:** Association between expression of TRAF6 and ubiquitin

**Clinical parameters**	**TRAF6**			
	**high**	**low**	**χ**^**2**^	**P**
ubiquitin			20.05	0.001
high	51(85.0%)	9(15.0%)		
low	18(42.9%)	24(57.1%)		

### Discussion

In healthy individuals, skeletal muscle metabolism requires a balance of anabolic and catabolic processes, resulting in a continuous renewal of muscle proteins without a net change in overall muscle mass. However, in cancer cachexia and other chronic illnesses, the muscle wasting were associated with the reduced rate of protein synthesis, increased protein degradation, or a combination of both contributes [13]. One common mechanism associated with skeletal muscle protein degradation in cancer cachexia is the activation of the adenosine triphosphate-dependent ubiquitin-proteasome proteolytic path way, this system plays a major role in muscle wasting [5,6]. The study showed muscle ubiquitin mRNA was hyper expressed in gastric cancer patients compared to controls [14], the ubiquitin-proteasome proteolytic system play important role in the pathogenesis of muscle protein hyper catabolism in cancer cachexia. To investigate the role of ubiquitin expression in the skeletal muscle of gastric cancer patients. We assessed the expression of ubiquitin in 29 control muscles and 102 patient muscles. Ubiquitin was significantly upregulated in muscle of gastric cancer compared with the control muscles. Over expression of ubiquitin in muscle of gastric cancer were associated with TNM stage and weight loss.

Skeletal muscle wasting is a major reason for morbidity and mortality in many chronic disease states, disuse conditions and aging. The ubiquitin-proteasome and autophagy-lysosomal systems are the two major proteolytic pathways involved in regulation of both physiological and pathological muscle wasting. The study demonstrate that the expression level of tumor necrosis factor (α) receptor adaptor protein 6 (TRAF6), a protein involved in receptor-mediated activation of several signaling pathways, is enhanced in skeletal muscle during atrophy [9,10]. To explore the relation of TRAF6 expression in the skeletal muscle of gastric cancer patients. We assessed the expression of TRAF6 in 29 control muscles and 102 patient muscles. TRAF6 was significantly upregulated in muscle of gastric cancer compared with the control muscles, Overexpression of TRAF6 in muscle of gastric cancer were associated with TNM stage, the level of serum albumin and percent of weight loss. The study showed overexpression of TRAF6 may play important role in gastric cancer cachexia.

Paul’s study discover that TRAF6 possesses E3 ubiquitin ligase activity causing lysine-63-linked polyubiquitination of target proteins. Muscle-wasting stimuli could up regulate the expression of TRAF6 and auto-ubiquitination. Muscle-specific depletion of TRAF6 preserves skeletal muscle mass in a mouse model of cancer cachexia or denervation. Inhibition of TRAF6 also blocks the expression of the components of the ubiquitin-proteasome system (UPS) and auto phagosome formation in atrophying skeletal muscle [15]. We also examined TRAF6 expression in skeletal muscle with gastric cancer and its correlation with ubiquitin status. We found a positive correlation between TRAF6 and ubiquitin expression, suggesting that TRAF6 may up regulates ubiquitin activity in cancer cachexia. While more investigations are required to understand its mechanisms of TRAF6 and ubiquitin in skeletal muscle. Correct the catabolic-anabolic imbalance is essential for the effective treatment of cancer cachexia.

## Competing interests

The authors declared that they have no competing interest.

## Authors' contributions

Y-SS and Z-YY design the study, Z-YQ, X-DX, and J-FH carried out the Real-time quantitative RT-PCR and Immunoblotting, Y-SS drafted the manuscript. All authors read and approved the final manuscript.
